# Cultivating the Bacterial Microbiota of *Populus* Roots

**DOI:** 10.1128/mSystems.01306-20

**Published:** 2021-06-22

**Authors:** Dana L. Carper, David J. Weston, Aditya Barde, Collin M. Timm, Tse-Yuan Lu, Leah H. Burdick, Sara S. Jawdy, Dawn M. Klingeman, Michael S. Robeson, Allison M. Veach, Melissa A. Cregger, Udaya C. Kalluri, Christopher W. Schadt, Mircea Podar, Mitchel J. Doktycz, Dale A. Pelletier

**Affiliations:** a Biosciences Division, Oak Ridge National Laboratory, Oak Ridge, Tennessee, USA; b Graduate School of Genome Science and Technology, University of Tennessee, Knoxville, Tennessee, USA; c Department of Biomedical Informatics, University of Arkansas for Medical Sciences, Little Rock, Arkansas, USA; d Department of Environmental Science and Ecology, University of Texas at San Antonio, San Antonio, Texas, USA; Purdue University

**Keywords:** poplar, plant microbiome, isolation, culture collection, 16S rRNA gene sequencing, bacterial isolation

## Abstract

The integral role of microbial communities in plant growth and health is now widely recognized, and, increasingly, the constituents of the microbiome are being defined. While phylogenetic surveys have revealed the taxa present in a microbiome and show that this composition can depend on, and respond to, environmental perturbations, the challenge shifts to determining why particular microbes are selected and how they collectively function in concert with their host. In this study, we targeted the isolation of representative bacterial strains from environmental samples of *Populus* roots using a direct plating approach and compared them to amplicon-based sequencing analysis of root samples. The resulting culture collection contains 3,211 unique isolates representing 10 classes, 18 orders, 45 families, and 120 genera from 6 phyla, based on 16S rRNA gene sequence analysis. The collection accounts for ∼50% of the natural community of plant-associated bacteria as determined by phylogenetic analysis. Additionally, a representative set of 553 had their genomes sequenced to facilitate functional analyses. The top sequence variants in the amplicon data, identified as Pseudomonas, had multiple representatives within the culture collection. We then explore a simplified microbiome, comprised of 10 strains representing abundant taxa from environmental samples, and tested for their ability to reproducibly colonize *Populus* root tissue. The 10-member simplified community was able to reproducibly colonize on *Populus* roots after 21 days, with some taxa found in surface-sterilized aboveground tissue. This study presents a comprehensive collection of bacteria isolated from *Populus* for use in exploring microbial function and community inoculation experiments to understand basic concepts of plant and environmental selection.

**IMPORTANCE** Microbial communities play an integral role in the health and survival of their plant hosts. Many studies have identified key members in these communities and led to the use of synthetic communities for elucidating their function; however, these studies are limited by the available cultured bacterial representatives. Here, we present a bacterial culture collection comprising 3,211 isolates that is representative of the root community of *Populus.* We then demonstrate the ability to examine underlying microbe-microbe interactions using a synthetic community approach. This culture collection will allow for the greater exploration of the microbial community function through targeted experimentation and manipulation.

## INTRODUCTION

*Populus* trees are cultivated worldwide for multiple industrial purposes, including the production of wood products, wind breaks, and, more recently, as biofuel feedstock (1–3). The health and survival of *Populus* are partially dependent on the consortia of microorganisms that make up its associated microbiome (4, 5). These organisms form a complex network of interactions both with the host and other microorganisms. The microbiome can enhance host tolerance to abiotic and biotic stresses such as drought (6–8), nutrient limitation (9, 10), pathogen presence (11), and increased salinity (12). For example, Doty et al. (13) demonstrated nitrogen fixation within wild poplar by a native microbiome, thereby mitigating nutrient limitation. Recently, it has been shown that some bacterial community members promote root colonization by the beneficial mycorrhizal fungus *Laccaria bicolor* (14). Bacterial communities in poplar trees are structured by plant compartment (root endosphere, rhizoplane, rhizosphere, stem, leaf endosphere, and leaf phyllosphere) (15, 16), soil characteristics (16–19), and host genotype (19, 20). While taxonomic studies, based on marker genes, have given an exploratory view of the microbial diversity (15, 21–23) and metagenomic studies have given insight into the possible functions of these communities (24, 25), it remains unclear which taxa perform specific changes in plant performance *in situ* and how these effects are integrated to result in microbiome-induced plant host benefits.

Cultivation-based approaches for microbial isolation have been used previously to identify microbial community members potentially beneficial to various plant species. Such studies have shown that specific bacterial isolates are capable of benefiting plants through multiple mechanisms. They also give genomic backgrounds, as many isolates are genome sequenced, for comparisons with metagenomic environmental data. An *Actinobacteria* strain isolated from wheat, for example, promotes host growth and water stress tolerance (7). Inoculation of maize plants with Azospirillum brasilense increases plant growth possibly due to increased nitrogen efficiency (26, 27). Similarly, *Pantoea* strains isolated from sugarcane and *Populus* enhance growth through production of indole acetic acid and by the microbe’s ability to solubilize phosphate (28, 29). Some Pseudomonas strains have documented antimicrobial compound production protecting black pepper and potato cultivars from pathogens (11, 30). In a study of Pseudomonas species isolated from *Populus deltoides*, it was determined that beneficial traits such as phosphate solubilization, denitrification, and growth promotion were increased in endosphere isolates relative to rhizosphere isolates, demonstrating the likelihood of niche specialization (31). These studies demonstrate the functionality of individual isolates and allow for extrapolation of functions within a larger phylogenetic context.

While inoculations with individual strains may give insight into a microbe’s contributions to host biology and allow for hypotheses regarding their possible function within a community, it is a major challenge to predict how microbial interactions will alter function in the context of a mixed bacterial community. To explore the mechanisms involved in community dynamics, some studies have shifted toward constructed or simplified communities. These constructed community experiments are carried out in laboratory settings using axenic host organisms allowing for precise definition of the bacterial communities and controlled environmental parameters. For example, constructed community approaches to explore plant microbiomes have been carried out in Arabidopsis thaliana (32–34), maize (35), and sugarcane (36). Studies of simplified communities in Arabidopsis thaliana have led to the discovery of multiple plant-excreted phytochemicals, such as salicylic acid and coumarins, that play roles in shaping the root microbiome (33, 34). Inoculation of axenic poplar plants with two poplar-associated microbial strains (Pseudomonas and *Paraburkholderia*) increased both root area and the photosynthetic capabilities of the host (37). When both strains were inoculated on the same plant an additive effect was seen, suggesting noncompetitive niches for each of the strains (37). Constructed communities, while elucidating underlying mechanisms of host selectivity, can also provide insight into complex microbe-microbe interactions that may be hard to detect in natural communities. For example, Niu et al. (35) found that removal of a single bacterial member, Enterobacter cloacae, led to a complete breakdown of community structure and the uncontrolled growth of Curtobacterium pusillum in maize roots. Their results showed a competitive interaction between multiple strains within the community, a consequence that would be hard to identify in more complex systems. Importantly, these studies have depended primarily on cultivable members of the plant microbiome. The well-described relationships between specific plants and microbial communities indicate extensive diversity in microbial potential and, thus, require diverse culture collections from which to draw representative microbial taxa (38).

In this work, we aimed to (i) generate a representative collection of bacterial isolates cultured from the rhizosphere and root endosphere of *Populus* trees, (ii) explore to what extent the culture collection represents the natural diversity observed using 16S rRNA gene sequencing, and (iii) demonstrate the ability to construct communities comprised of bacterial isolates from this collection that are capable of reproducibly colonizing and benefiting host poplar plants. With this collection, we will be able to expand greatly the knowledge of plant-microbe interactions. The large number of isolates allows for the construction of many synthetic communities that range from simple to complex in order to gain a greater understanding of community assembly. These isolates also allow for the exploration of the underlying genetics involved in plant-microbe interactions.

## RESULTS

### Isolation of bacteria from *Populus* roots and rhizosphere.

**FIG 1:**
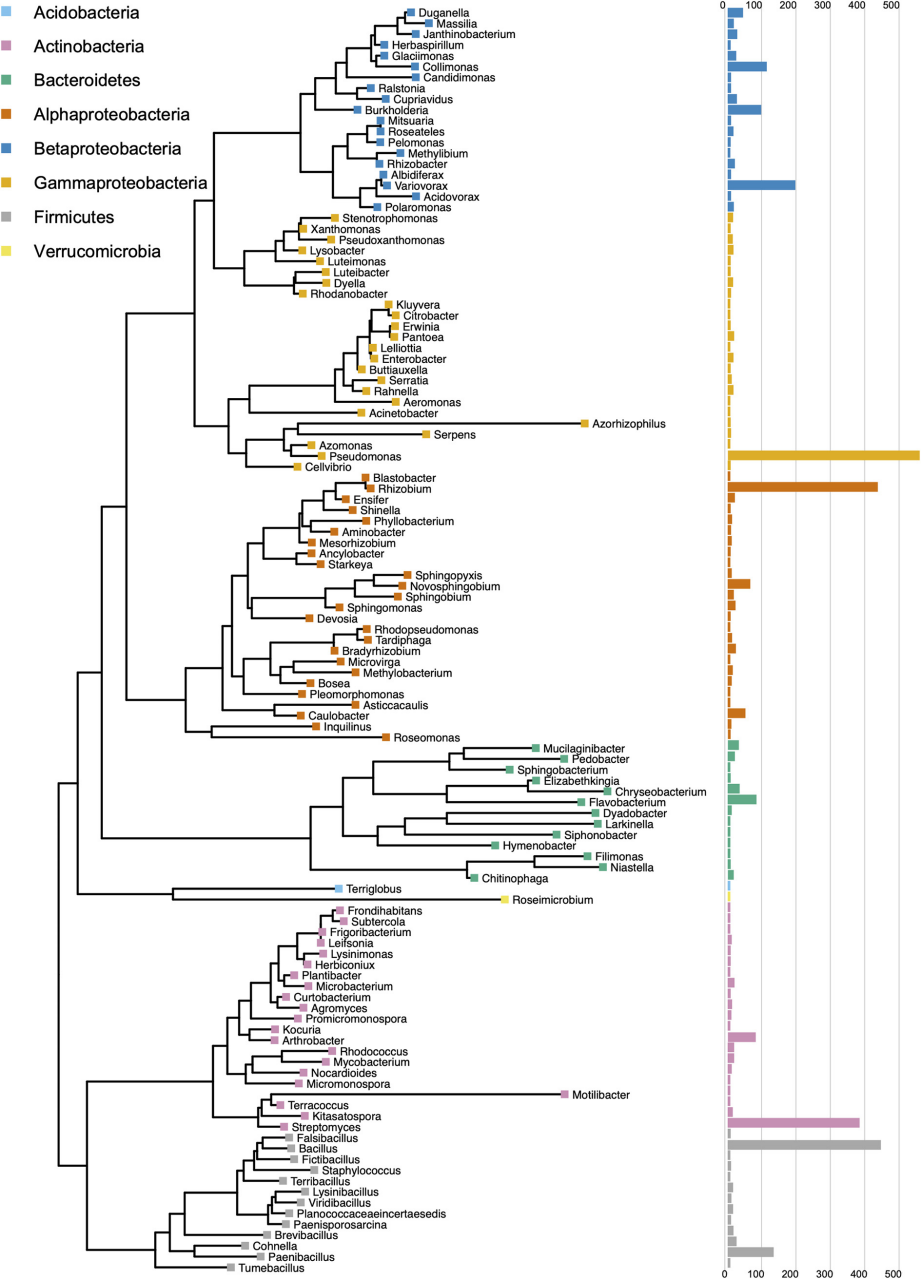
Phylogenetic tree created from 16S rRNA sequencing of all isolates consolidated into genera. Tip points are colored by the bacterial phylum/class (*Proteobacteria*) to which they belong. The bar graph corresponds to the number of isolates in the collection in that genus.

**FIG 2:**
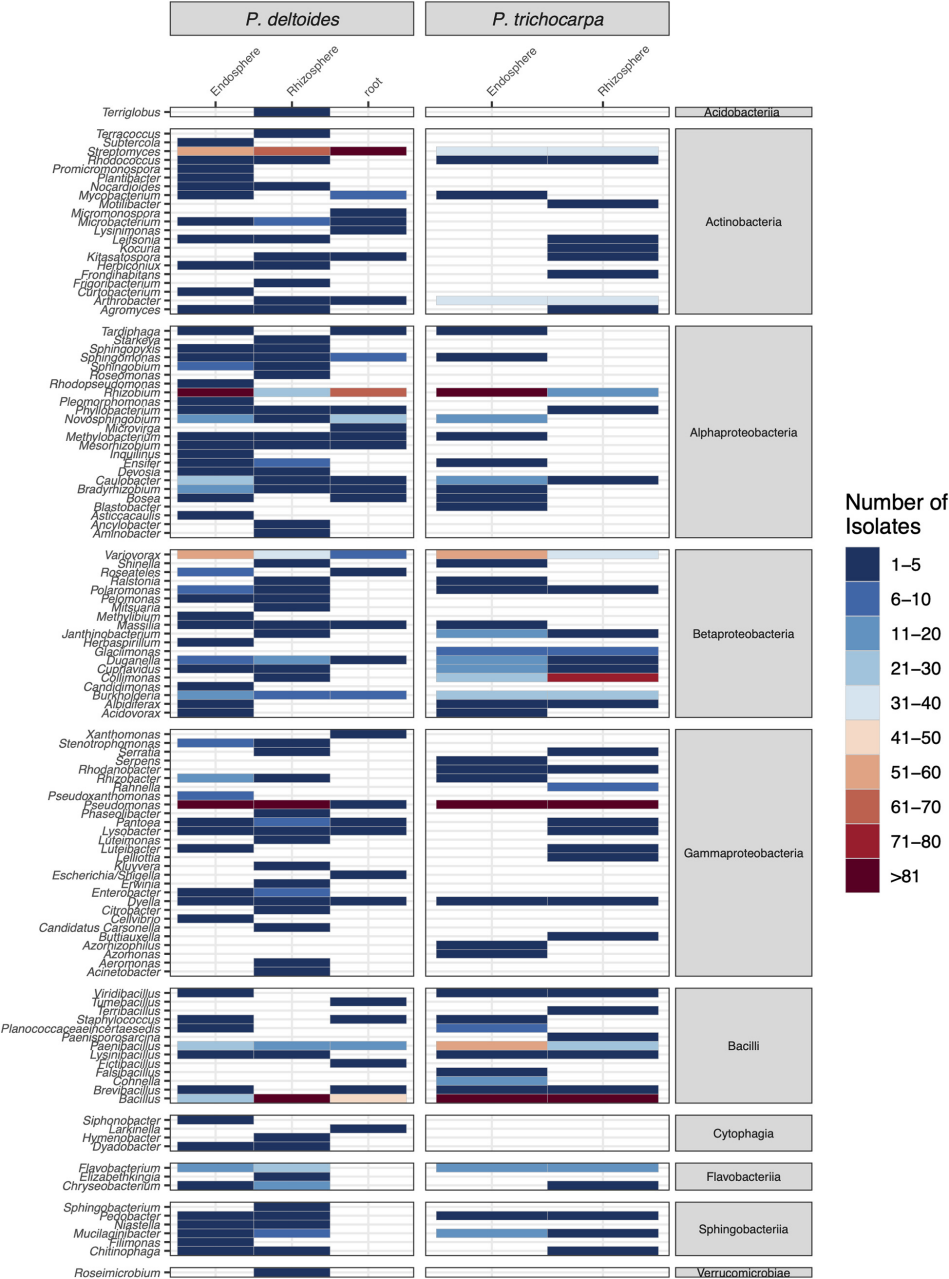
Heatmap showing the number of isolates from root endosphere and rhizosphere. The genus to which the isolates belong is listed along the *y* axis. The larger groupings are the tree species across the top and the bacterial class along the right side.

There are currently limited bacterial isolates from the *Populus* root microbiome for use as reference genomes for metagenomic comparisons, comparative bacterial genomics, and synthetic community construction. To increase these resources, we isolated a large (3,211) bacterial culture collection from fine roots (<2 mm) of two *Populus* species. We sampled the rhizosphere, which includes soil adhered tightly to the roots, rhizoplane organisms, the root endosphere, and macerated surface-sterilized roots, in an effort to capture as much diversity as possible. Dilution plating and subsequent colony isolation resulted in 3,208 pure bacterial cultures (see Table S1 in the supplemental material) from *Populus deltoides* and *Populus trichocarpa* roots and rhizosphere, sampled across two *Populus* common garden sites (39) and natural environments in Tennessee, Georgia, Oregon, and North Carolina between 2011 and 2018. In addition, three isolates were obtained by single-cell flow cytometry sorting on agar medium in order to begin to access less abundant members of the *Populus* microbiome. The majority of the isolates (87%) were cultured using R2A media (Table S1) (40), although various other media and root extracts were also used to increase strain diversity. The diversity of the microbial isolates was assessed based on 16S rRNA gene sequence and determined to include bacterial isolates representing 10 classes, 18 orders, 45 families, and 120 genera from 6 phyla: *Acidobacteria*, *Actinobacteria*, *Bacteroidetes*, *Firmicutes*, *Proteobacteria*, and *Verrucomicrobia* (Fig. 1, Table 1; see also Table S1). The greatest number of isolates are from 5 genera, Pseudomonas (18.2%, 584), *Bacillus* (13.7%, 439), *Rhizobium* (13.4%, 430), *Streptomyces* (11.8%, 377), and *Variovorax* (5.9%, 190). The large number of bacteria from these groups is likely the result of their abundance within the microbiome, their presence in the majority of samples, and culture conditions that favor their growth, as many of these genera are known to be more readily cultivated in a laboratory setting. At 97% sequence similarity, we estimate that 464 distinct species exist within the culture collection, with the most species coming from the genera *Paenibacillus* (9.7%, 45), followed by *Bacillus* (7.8%, 36), Pseudomonas (6.7%, 31), *Streptomyces* (5.8%, 27), and *Rhizobium* (5.6%, 26). The majority of the isolates were cultured from the surface-sterilized root endosphere (50%) followed by rhizosphere (37.3%) and unsterilized root tissues (12.7%) of *Populus* (Fig. 2). Although more individual isolates were cultured from the root endosphere, a greater number of unique genera was isolated from the rhizosphere (91 genera) versus root endosphere (83 genera). The genera with the greatest number of isolates (Pseudomonas, *Bacillus*, *Rhizobium*, *Streptomyces*, and *Variovorax*) were present across both host species (*P. deltoides* and *P. trichocarpa*) and across all sampled trees, while other genera showed host species specificity (Fig. 2). Strains from the genera *Collimonas* (*Betaproteobacteria*) and *Arthrobacter* (*Actinobacteria*), while not exclusive, were primarily and more frequently isolated from *P. trichocarpa* (*Collimonas*, 1 strain from *P. deltoides* versus 106 strains from six out of 11 *P. trichocarpa* trees; *Arthrobacter*, 3 strains from 3 of 41 *P. deltoides* trees versus 71 strains from 9 out of 11 *P. trichocarpa* trees). The classes *Cytophagia*, *Verrucomicrobiae*, and *Acidobacteria* were isolated only from *P. deltoides* trees, although only a few strains from *Cytophagia* and 1 strain each from *Verrucomicrobiae* (41) and *Acidobacteria* (42) are present within the collection. Host species may be only one contributing factor, as climate and soil type could also influence the distribution of isolates. Some genera were more abundant in either the root endosphere or rhizosphere. *Streptomyces*, although present in both the root endosphere and rhizosphere, had the most strains isolated from the non-surface-sterilized root samples (Fig. 2). A few genera (*Microbacterium*, *Tardiphaga*, and *Bosea*) were cultured only from the root endosphere and unsterilized root samples, but only a few strains are present within the collection (Fig. 2).

**TABLE 1:** Number of isolates in culture collection identified by 16S rRNA taxonomic analysis^*a*^

Putative ID	Count
*Acidobacteria*	1
*Acidobacteriia*	1
*Actinobacteria*	530
*Actinobacteria*	503
*Actinobacteridae*	27
*Bacteroidetes*	168
*Cytophagia*	8
*Flavobacteria*	107
*Sphingobacteria*	53
*Firmicutes*	628
*Bacilli*	628
*Proteobacteria*	1,883
*Alphaproteobacteria*	645
*Betaproteobacteria*	538
*Gammaproteobacteria*	700
*Verrucomicrobia*	1
*Verrucomicrobiae*	1

a Grey indicates phylum-level identification, and no shading indicates class level.

10.1128/mSystems.01306-20.7 TABLE S1 Table of all isolates listed by strain designation (id) with associated metadata of tree id, tree species, isolation year, sample collection location, isolation source, isolation media, taxonomic classification, and 16S rRNA gene sequence. Download Table S1, XLSX file, 0.8 MB. Copyright © 2021 Carper et al. 2021 Carper et al. https://creativecommons.org/licenses/by/4.0/ This content is distributed under the terms of the Creative Commons Attribution 4.0 International license.

For two genera, *Rhizobium* and *Variovorax*, more isolates were cultured from the root endosphere samples compared to rhizosphere (Fig. S1). Based on RDP classification, the *Variovorax* isolates, when assigned against the RDP database, are found to belong to 5 different species, *V. boronicumulans*, *V. ginsengisoli*, *V. guangxiensis*, V. paradoxus, and *V. soli* (Table S2). Even though more isolates were cultured from the root endosphere, four of the species listed above were isolated from both root endosphere and rhizosphere. The only notable exceptions are the isolates identified as Variovorax soli, which were exclusively found in the rhizosphere or non-surface-sterilized root samples, suggesting that these isolates remain on the outer surfaces of the plants. Similar to *Variovorax*, the *Rhizobium* isolates had many species shared across both root endosphere and rhizosphere (Table S3). This suggests that isolates within a species have specific adaptations for survival in the different root regions. One species of *Rhizobium* identified in the isolate collection as being in the rhizosphere was *R. paranaense*. This species is commonly found in beans as a nitrogen-fixing symbiont (43). A species only identified in the endosphere, *R. cellulosilyticum*, was previously isolated from sawdust of *Populus alba* and generally has polysaccharide-hydrolyzing ability, suggesting a mechanism for its entry into the endosphere compartment (44); however, further studies are needed to confirm root endosphere colonization mechanisms.

10.1128/mSystems.01306-20.1 FIG S1 Comparisons in the number of root endosphere and rhizosphere strains in genera with more than 10 isolates. Download FIG S1, TIF file, 0.4 MB. Copyright © 2021 Carper et al. 2021 Carper et al. https://creativecommons.org/licenses/by/4.0/ This content is distributed under the terms of the Creative Commons Attribution 4.0 International license.

10.1128/mSystems.01306-20.8 TABLE S2 *Variovorax* isolates with associated metadata of tree id, tree species, isolation year, sample collection location, isolation source, RDP taxonomic match, taxonomic classification, and IMG genome IDs for genome sequenced strains. Download Table S2, XLSX file, 0.02 MB. Copyright © 2021 Carper et al. 2021 Carper et al. https://creativecommons.org/licenses/by/4.0/ This content is distributed under the terms of the Creative Commons Attribution 4.0 International license.

10.1128/mSystems.01306-20.9 TABLE S3 *Rhizobium* isolates with associated metadata of tree id, tree species, isolation year, sample collection location, isolation source, RDP taxonomic match, taxonomic classification, and IMG genome IDs for genome sequenced strains. Download Table S3, XLSX file, 0.04 MB. Copyright © 2021 Carper et al. 2021 Carper et al. https://creativecommons.org/licenses/by/4.0/ This content is distributed under the terms of the Creative Commons Attribution 4.0 International license.

### Comparison of isolate 16S rRNA gene sequence with phylotyping data.

**FIG 3:**
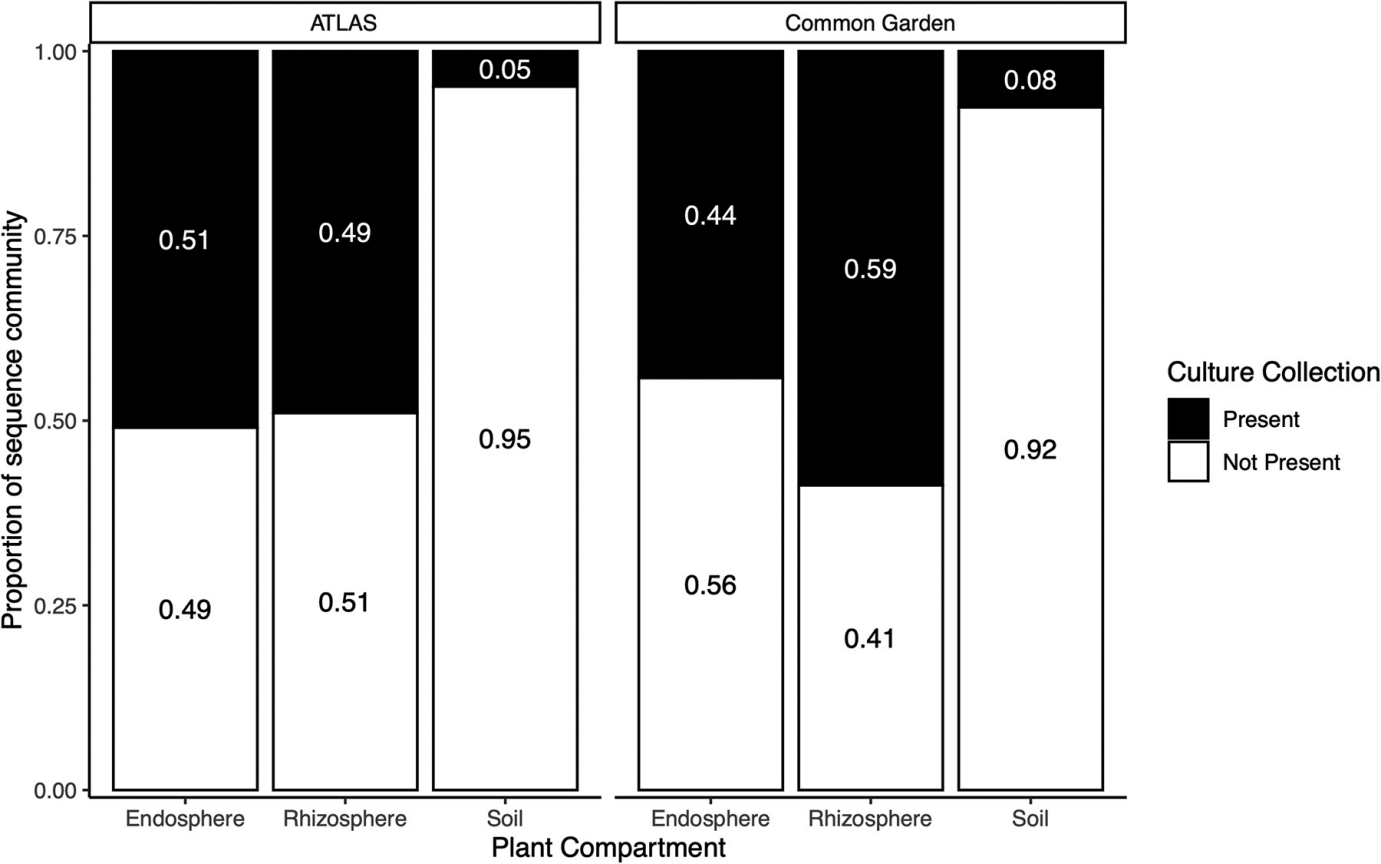
Proportion of 16S sequencing data, from previous poplar studies, that is present within the culture collection from the rhizosphere, endosphere, and soil.

**FIG 4:**
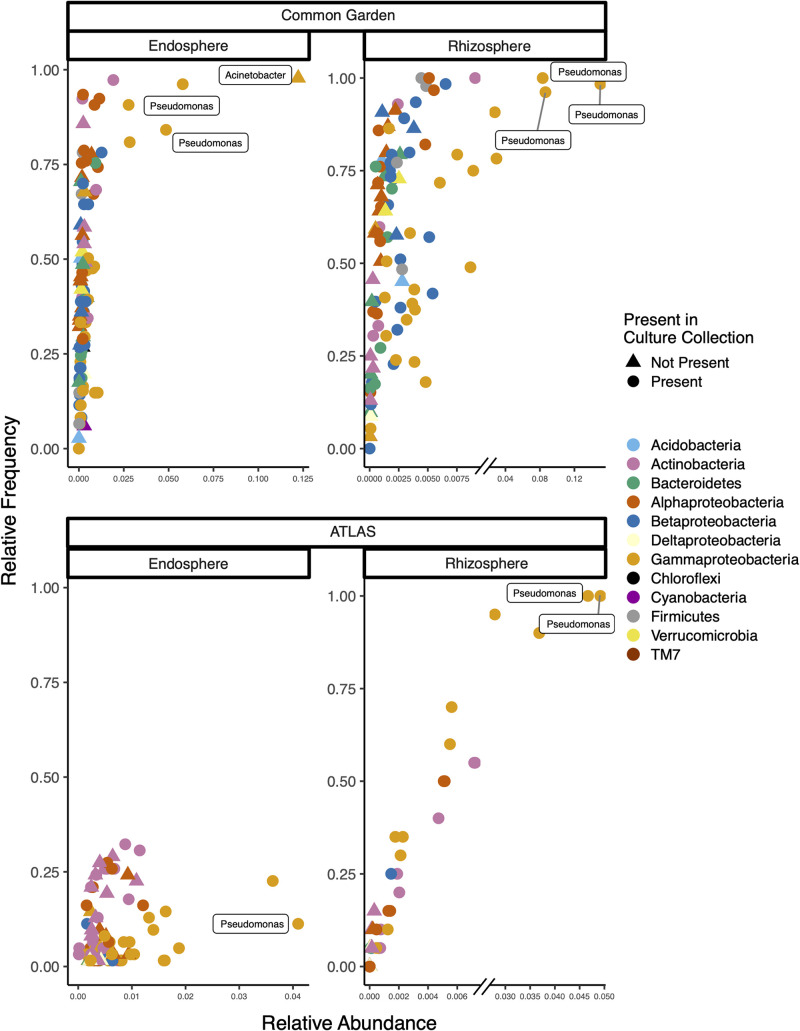
Relative abundance of top 100 sequence variants from previous poplar microbiome studies plotted against their relative frequency (how many trees they were found in). Color indicates the phylogenetic class to which the sequence variant was identified.

To explore to what extent the bacterial isolates reflect the natural diversity of the *Populus* microbiota, we compared 16S gene sequences of culture collection isolates with rRNA gene survey data from root endosphere, rhizosphere, and bulk soil samples. We used two gene survey studies. The first study contained samples collected from *Populus trichocarpa* trees grown in common gardens at Corvallis, OR, and Clatskanie, OR (designated the common garden study). These samples were also utilized for isolation in the collection. The second study was a previously published data set available for *P. deltoides* grown at a site in Knoxville, TN (designated the Atlas study) (15). Overall, 50% of the sequences found in the amplicon data (root endosphere and rhizosphere) had a representative within the culture collection compared at 97% identity levels (Fig. 3). The common garden study had less cultured representatives in the root endosphere (44.2% ± 19.7%) compared to the rhizosphere data (58.8% ± 25.3%). The Atlas study had slightly more cultured representatives in the root endosphere (51% ± 30.3%) than the rhizosphere (49% ± 15.2%). Very little of the culture collection was matched in the bulk soil samples from either the Atlas (4.7% ± 2.1%) or the common garden (7.5% ± 2.6%) studies, highlighting the selectivity and recruitment by plants for organisms in the environment. The top sequence variants in the root endosphere and rhizosphere were identified as Pseudomonas species (Fig. 4). In studies and both rhizosphere and root endosphere, the majority of the top sequence variants had a match to the culture collection, with one notable exception. In the common garden study root endosphere samples, the top sequence variant that was present in almost all samples was identified as an Acinetobacter species. Although the culture collection does contain an Acinetobacter species, the sequence variant was only 95% genetically similar based on 16S rRNA. The sequence variant from the common garden sample had 100% sequence identity to Acinetobacter johnsonii species based on a BLAST search. A phylogenetic analysis of Acinetobacter species, including the sequence variant and the isolated strain from the culture collection, show the sequence variant grouped with Acinetobacter johnsonii species while the culture collection isolate grouped with Acinetobacter calcoaceticus (Fig. S2).

10.1128/mSystems.01306-20.2 FIG S2 Cladogram of Acinetobacter species. The one strain from the isolate collection (red) and the most abundant sequence variant (blue) in the common garden root endosphere. Node labels are SH-aLRT support (%)/ultrafastbootstrap support (%). Download FIG S2, TIF file, 1.4 MB. Copyright © 2021 Carper et al. 2021 Carper et al. https://creativecommons.org/licenses/by/4.0/ This content is distributed under the terms of the Creative Commons Attribution 4.0 International license.

### Simplified, representative community behavior on axenic *Populus*.

**FIG 5:**
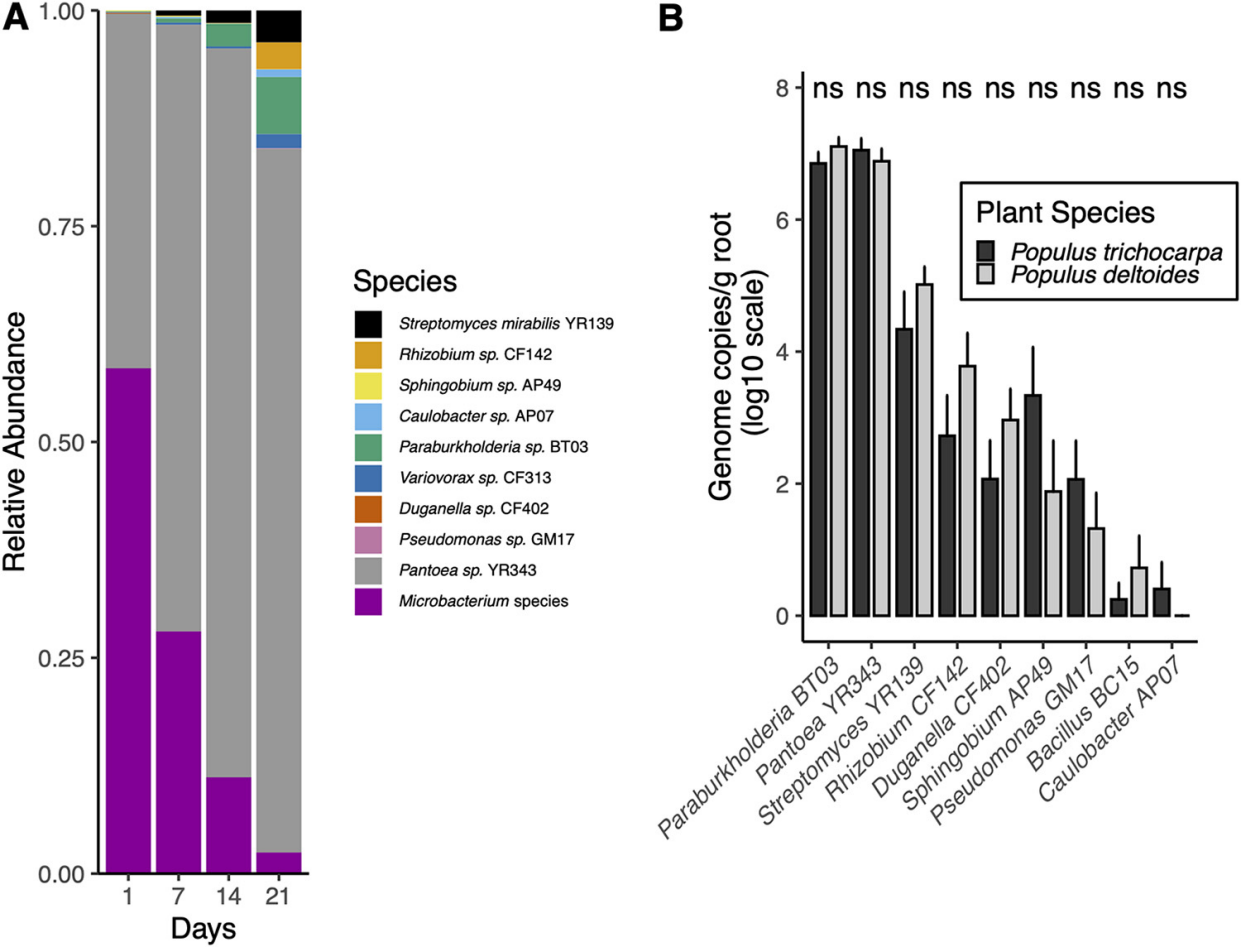
Quantification of 10-member bacterial community strains on *Populus* root tissue. (A) Taxonomic plots of strain relative abundance based on 16S rRNA gene amplicon sequence of 10-member bacterial community on *Populus trichocarpa* root tissues and monitored over time. (B) Strain-specific qPCR results from each of the 10-member strains inoculated in a community on *Populus trichocarpa* and *Populus deltoides* roots with significant differences of colonization between species noted. Ns, not significant.

To investigate if isolates from the collection could establish a reproducible community, a simplified 10-member community was created using isolates from *Populus deltoides*. The community was created to resemble a natural poplar bacterial community at the phylum taxonomic rank (15), consisting of genome-sequenced organisms to allow us to uniquely assign genetic material to organisms to test hypotheses about functionality of individuals in a community; this community will be referenced as PD10. The 10 members included Streptomyces mirabilis YR139 (*Actinobacteria*), *Bacillus* sp. strain BC15 (*Firmicutes*), *Sphingobium* sp. strain AP49 (*Alphaproteobacteria*), *Caulobacter* sp. strain AP07 (*Alphaproteobacteria*), *Rhizobium* sp. strain CF142 (*Alphaproteobacteria*), *Paraburkholderia* sp. strain BT03 (*Betaproteobacteria*), *Variovorax* sp. strain CF313 (*Betaproteobacteria*), *Duganella* sp. strain CF402 (*Betaproteobacteria*), Pseudomonas sp. strain GM17 (*Gammaproteobacteria*), and *Pantoea* sp. strain YR343 (*Gammaproteobacteria*). Based on KEGG orthology (KO), which looks at the functional potential contained in genomes, this phylogenetically and functionally diverse community represents the majority of the functional potential present in the genome-sequenced strains from our culture collection. Specifically, of the 9,886 KO terms detected in the *P. deltoides* root endosphere metagenome (45), the PD10 community represented 4,621 (47%) of the KO terms. Interestingly, we detected 121 KO terms unique in the PD10 genomes, likely due to the ability to resolve low-abundance genes in microbiome samples (Fig. S3). The PD10 community was used in three experiments on axenic *Populus* roots. The first experiment examined the PD10 members over four time points after inoculation. Initial poplar roots were dominated by a *Microbacterium* species that was not part of the PD10 community, suggesting endogenous origin (Fig. 5A). The *Microbacterium* species was also present in the uninoculated control samples (Fig. S4). The poplar root environment supported a final community of 8 of the 10 members, although the community was dominated by *Pantoea* sp. strain YR343 (80% relative abundance).

10.1128/mSystems.01306-20.3 FIG S3 Comparison of native microbial metagenome to 10 member constructed community. (A) Data source and phylum-level alignment of metagenomics reads. Inset shows how addition of additional organisms increases representation of the native metagenome by PFAM, KO, or TIGRFAM annotations. (B) Venn diagram showing overlap between constructed community (blue) and wild metagenome (orange). The black circle represents the core KO terms present in all 10 microbial isolate genomes. Download FIG S3, TIF file, 0.7 MB. Copyright © 2021 Carper et al. 2021 Carper et al. https://creativecommons.org/licenses/by/4.0/ This content is distributed under the terms of the Creative Commons Attribution 4.0 International license.

10.1128/mSystems.01306-20.4 FIG S4 Taxonomic plots based on 16S rRNA gene amplicon sequence of uninoculated and PD10 inoculated *Populus trichocarpa* root tissues and monitored over time. Download FIG S4, TIF file, 0.7 MB. Copyright © 2021 Carper et al. 2021 Carper et al. https://creativecommons.org/licenses/by/4.0/ This content is distributed under the terms of the Creative Commons Attribution 4.0 International license.

The second experiment was single inoculations of each of the PD10 members onto axenic *Populus* roots, and colonization of roots was assessed by counting CFU for each strain individually. The number of CFU ranged from 10^2.5^ CFU/g of root by *Caulobacter* AP07 to 10^7.7^ CFU/g of root by *Paraburkholderia* sp. strain BT03 (Fig. S5). Both *Paraburkholderia* sp. strain BT03 and *Pantoea* sp. strain YR343 had greater colonization rates than all of the other strains. In the third experiment, the PD10 community was inoculated onto axenic *Populus deltoides* and *Populus trichocarpa* roots, and the final community was evaluated using quantitative PCR (qPCR). Similarly, to the single inoculations, when inoculated in a community, *Paraburkholderia* sp. strain BT03 and *Pantoea* sp. strain YR343 had the highest colonization rate assessed through qPCR independent of the host species (Fig. 5B). Three strains, *Caulobacter* sp. strain AP07, *Bacillus* sp. strain BC15, and Pseudomonas sp. strain GM17, decreased in colonization potential when inoculated in a community compared to single-strain inoculations.

10.1128/mSystems.01306-20.5 FIG S5 Colony-forming counts of individual strains after inoculation on *Populus trichocarpa* roots. X indicates data are not available. Download FIG S5, TIF file, 0.2 MB. Copyright © 2021 Carper et al. 2021 Carper et al. https://creativecommons.org/licenses/by/4.0/ This content is distributed under the terms of the Creative Commons Attribution 4.0 International license.

## DISCUSSION

Microbial communities associated with plants are extremely complex in taxonomic and functional diversity as well as spatial and temporal organization. While taxonomic marker gene and metagenomic studies can give us insights into community structure and functional potential, understanding the roles of individual members in the community is still a grand challenge. To gain a better understanding of microbial community assembly and functioning, we have amassed a culture collection of 3,211 unique bacterial isolates, of which 553 have been genome sequenced, and 209 genome-sequenced isolates are newly released in this paper (41, 42, 45–53) (Table S4). These isolates derive from the rhizosphere and root endosphere of natural populations or field-grown (common garden-planted) poplar trees. We demonstrate that the culture collection represents a large fraction of the natural bacterial community, as assessed by comparison to 16S rRNA amplicon data. We also demonstrate the use of the isolates in simplified communities to understand some of the complex interactions between microbes.

10.1128/mSystems.01306-20.10 TABLE S4 Table of genome sequenced strains within the isolate collection with hyperlinks to NCBI and GenBank accession numbers. The table also contains metadata on sequencing center, assembly method, release data, sequencing method, gene counts scaffold counts, and GC content. Download Table S4, XLSX file, 0.1 MB. Copyright © 2021 Carper et al. 2021 Carper et al. https://creativecommons.org/licenses/by/4.0/ This content is distributed under the terms of the Creative Commons Attribution 4.0 International license.

We found that many genera (Pseudomonas, *Bacillus*, *Rhizobium*, *Streptomyces*, and *Variovorax*) were ubiquitous across host species and plant organ, and these tended to be the genera with the most isolates. Overall, this is not surprising, as natural poplar communities have a large overlap in community composition between host species and plant organ (15). However, both *Variovorax* and *Rhizobium* had more isolates cultivated from the endosphere than rhizosphere. A more in-depth analysis of these two genera demonstrates that many species were found in both root endosphere and rhizosphere, suggesting strain-level variation is responsible for the difference in colonization, although more research is needed to identify the genes within a given strain involved in root colonization. *Pseudomonadaceae* commonly dominate the tissues of multiple plant species (54), and Pseudomonas isolates have the most representatives within the culture collection, with 584 isolates. These Pseudomonas isolates represent potentially 31 different species and multiple strains within those species, showcasing the large amount of diversity within this genus. A few studies have explored the diversity within the Pseudomonas isolates more thoroughly (31, 46, 55). These show the diversity within only a few of our strains and demonstrate that the metabolic potential of a strain even within a single species is based on the compartment from which it was isolated (31). Similarly, a study using leaves and branches of poplar plants showed that the majority of isolates they obtained were identified as Pseudomonas (20), although the same study also recovered many isolates identified as *Sphingomonas*, a genus that has few representatives within our culture collection. This suggests that it is a more abundant member in leaves (20). Previous studies using Pseudomonas isolates from our collection have shown plant beneficial traits, such as growth promotion and phosphate solubilization (31, 37). Some other genera of isolates were primarily found in a specific host species or root region. *Glaciimonas* strains, which were exclusively isolated from *P. trichocarpa* in our collection, have representatives that have been isolated primarily from cold environments such as glaciers (56, 57). The presence of the *Glaciimonas* strains in *P. trichocarpa* could be the result of their geographic distribution along the west coast of Canada to southern Alaska, in generally colder climates; however, differences in microclimates, soil type, and other site characteristics could be the cause of this pattern. Other genera seemed more abundant in specific root endosphere compared to the rhizosphere, which is consistent with culture-independent studies of many plant species (15, 58, 59). Interestingly, *Variovorax*, *Rhizobium*, and *Novosphingobium* had more isolates cultured from the root endosphere than rhizosphere. This is consistent with a recent amplicon study that found those same three genera are enriched in the root endosphere of field-grown *Populus* trees (58).

Many plant-microbe studies have compared isolation-based methods to community sequencing approaches with various amounts of overlap between the two methods. For example, a study of the bacterial community in the xylem of olive trees found 41.2% of the genera that were detected using next-generation sequencing were found in their culture collection (60). A similar study exploring the bacterial community in the leaves of *Passiflora incarnata* found the number of bacterial species recovered represented 20% of the sequencing-based community (61). Similarly, a study of the rhizosphere of tomato plants found 22% overlap from their culture-dependent and culture-independent approaches (62). A study on the aerial components of poplar trees using both culturing and molecular fingerprinting identified 513 bacterial isolates with 27 distinct genera. Similar to the present study, they found the isolates comprised 51% of the genera detected with molecular fingerprinting approaches (20). In our study, roughly 50% (44% to 59%) of our plant-associated sequence-based communities have a cultured representative within the collection. When the soil samples from the same sites of the plant-associated communities were examined, only 5 to 7.5% of the sequence variants had a match to the culture collection. This indicates that the culture collection is comprised of microbial strains that are plant associated. The proportion of representatives in the rhizosphere and root endosphere is similar to that observed in the olive xylem (60) and poplar leaf (20) studies, although any differences could be the result of how representativeness was calculated. In the olive xylem (60) and tomato rhizosphere (62) studies, representativeness was calculated based solely on the genus of the organism. Our calculation compares individual sequences from the amplicon data to the 16S rRNA of the culture collection using a sequence similarity metric. This results in many sequences having 100% identity to multiple strains (e.g., Pseudomonas), as the 16S amplicon data provide only a short region for matching. This has been identified as one of the problems with comparing culture-dependent and culture-independent data sets (63). Here, we are reporting that our culture collection contains a representative that is phylogenetically similar to the amplicon data, not the exact strain, and that the top amplicons have one or multiple representatives (e.g., Pseudomonas strains). The collection presented here could have higher representation than other studies due to the large number of isolates that were cultivated and that multiple tree species and locations were used for cultivation. Most other bacterial studies have reported between 100 and 531 strains cultured (20, 60–62), whereas our collection contains 3,211 isolates, possibly providing a better cultured representation of the plant microbiome.

Despite the large number of isolates in the culture collection, there are some taxa that are present in amplicon data but not the collection. Specifically, the top sequence variant within the common garden root endosphere samples remained elusive. The sequence variant was identified as an Acinetobacter species closely related to Acinetobacter johnsonii; however, it was only 95% genetically similar to the Acinetobacter strain within the culture collection, which is genetically similar to Acinetobacter calcoaceticus. Acinetobacter species are widely recognized for causing infections in humans (64) but are commonly found in soil and water (65). Acinetobacter species isolated from soils have documented phosphate solubilization potential (66). Acinetobacter strains in association with plants have demonstrated growth-promoting ability, and shoot tissues have shown phosphate accumulation (67, 68). A strain of Acinetobacter johnsonii isolated from surface-sterilized beet roots showed growth-promoting effects on seedlings, and plants had increased absorption of nitrogen, phosphorus, and potassium from the soil (69). Within the top 100 sequence variants in the amplicon studies, a few major groups are missing representatives within the culture collection. The majority of the groups missing are those that have very few culture representatives in general. For example, we have not captured any TM7 representatives; however, only a few members of this phylum have recently been isolated (70–72). It was discovered that these microbes survive by interacting with a bacterial host species, complicating the cultivation process. Another group is *Cyanobacteria*, which can be difficult due to their slow growth, allowing for contamination by other faster-growing bacterial members (73). Current efforts are underway to target the Acinetobacter species that is present in a high proportion in the common garden samples and other uncultivated groups using newly developed techniques (42, 74).

The simplified 10-member community demonstrates the reproducibility of a constructed community in the roots of poplar. Surprisingly, the poplar roots started on day 1, 24 h after inoculation with the simplified community, with a high relative abundance of a *Microbacterium* species that was not part of the initial inoculum. It was also present in uninoculated controls, suggesting either endogenous origin of this bacteria or contamination. A study of the leaves and branches in field-grown poplar found over 47% of their isolates belong to *Microbacteriaceae*, indicating this is a common component in natural poplar microbiomes and likely of endogenous origins (20). The PD10 community displaces the *Microbacterium* species over time, with very little of this initially dominant microbe present after 21 days. The amplicon data revealed dominance by *Pantoea* sp. strain YR343, with a final relative abundance of ∼80% (Fig. 5A). Interestingly, using qPCR, both *Pantoea* sp. strain YR343 and *Paraburholderia* sp. strain BT03 reached similar high colonization levels (Fig. 5B). Differences in the two approaches could be the results of primer bias. qPCR was based on strain-specific primers giving a more accurate representation of the community, while the amplicon data used universal primers that could favor certain species over another. *Paraburkholderia* sp. strain BT03 colonizes poplar roots extremely well based on the qPCR results presented, suggesting that this bacterium thrives in a host-associated environment. Many species of *Paraburkholderia* are plant-associated beneficial microbes, some with nitrogen-fixing capabilities (75, 76). The simplified community had 8 of the 10 final members within the roots of poplar plants based on amplicon data, which is likely due to the high diversity in exudates and carbon substrates within the roots (77–79). Similarly, the complex spatial niches within the root are likely to support a more diverse community of microbes (80). The low colonization potential of some strains (*Bacillus* sp. strain BC15 and *Caulobacter* sp. strain AP07) is likely the result of competitive exclusion, where strains (*Paraburkholderia* sp. strain BT03 and *Pantoea* sp. strain YR343) are able to quickly colonize and, with increased growth, exclude some of the slower-growing/colonizing strains. However, it is possible that the potentially endogenous *Microbacterium* also contributed to the exclusion of strains through competition, although this seems unlikely given how fast *Microbacterium* decreases in abundance. Unlike natural communities, the simplified community was not dominated by Pseudomonas. This could be due to multiple reasons, including that only a single strain of Pseudomonas was used for this experimentation, whereas 548 strains have been isolated from natural roots, meaning another strain could be a better colonizer. Another potential difference could be the age of the cuttings used in the simplified community experiments. The cuttings were only 6 weeks old, while the natural samples were adult trees, as the age of the plants has been shown previously to influence the microbial communities (81–83). More research using simplified communities is needed to understand the factors governing assembly, including host genetics and the environment.

### Conclusions.

Here, we present a culture collection of 3,211 bacterial isolates cultured from the *Populus* root endosphere and rhizosphere. This is the most comprehensive collection of *Populus* isolates reported. It represents a large diversity of the natural *Populus* bacterial microbiome and will allow for in-depth analysis of plant-microbe interactions. With such an expansive collection, it will be possible to further elucidate the genetic potential within a microbiome. This collection could lead to a better understanding of the bacterial genetics involved in host-microbe interactions and elucidate the mechanisms by which microbes can assist plant hosts. Having a diverse collection of cultures will allow researchers to create larger synthetic communities with a known set of functional genes within controlled experimental manipulations to elucidate individual microbial roles and community adaptations. Overall, this collection will expand knowledge of plant-microbe interactions.

## MATERIALS AND METHODS

### Isolate collection.

The goal was to establish a diverse collection of bacterial isolates from the *Populus* root endosphere and rhizosphere microbiota. To capture as much diversity as possible, we utilized a wide array of root samples from natural and agricultural systems and different geographic locations, which are further detailed below. Bacterial strains were isolated through dilution plating on designated agar media (see Table S1 in the supplemental material). Roots from *Populus deltoides* or *P. trichocarpa* were sampled between 2009 and 2018 from Tennessee (29 trees), Georgia (12 trees), Oregon (33 trees), and North Carolina (11 trees) (Table S1). The genome-wide association study (GWAS) was of common gardens located near Corvallis, OR, and Clatskine, OR. These plantations consist of ∼1,100 individual genotypes of *Populus trichocarpa* replicated in three blocks, as described by Evans et al. (39). The Corvallis GWAS site lies on a sandy to gravelly loam mollisols in the Camas and Newberg series. The Clatskanie site lies on a silt loam entisol in the Wauna series. The Bellville, Georgia, testbed sites consist of plantations of wild-type and RNA knockdown lines of *Populus deltoides* and lies on loamy ultisols in the Irvington series. The Caney Fork and Yadkin Rivers populations consist of wild *Populus deltoides* that were sampled at 10 to 15 locations along the watershed of each river in Dekalb and Smith counties in Tennessee (Caney Fork) and Davie and Yadkin counties in North Carolina. Soil characteristics at these sites vary and were described previously (16, 18). The root samples were packed on wet ice in the field and sent to Oak Ridge National Laboratory for processing. Fine roots (<2 mm) were excised and processed as described previously (16, 84). To collect the rhizosphere bacteria, the roots were washed 5 times with sterile water, and the rhizosphere samples were collected from serial dilution of the washes. To collect root endosphere bacteria, the roots were then surface sterilized by a 30-s incubation in 95% ethanol, 3-min incubation in 5% NaOCl, and then 6 washes with sterile water (16). Following sterilization, roots were macerated in 10 ml of MgSO_4_ (10 mM) and serially diluted to cultivate endosphere microbes. Nonsterilized roots of some samples were macerated and plated and are referred to as root (Table S1). Cultures were isolated through 3 rounds of restreaking on agar medium. For all strains, 16S rRNA Sanger sequencing was carried out using universal primers (27f-1492r) (Table S1). Strains for genome sequencing were first picked based on phylogenetic diversity, followed by tree species and root region (endosphere and rhizosphere), with emphasis on strains from the root endosphere. Genome sequencing of strains (Table S2) was primarily carried out at the Joint Genome Institute (JGI; Walnut Creek, CA, USA) (https://jgi.doe.gov/), and annotations were carried out using the DOE-JGI Microbial Genome Annotation Pipeline (MGAP v.4) (85), as previously described (45, 46). The 16S rRNA sequences were input into the CD-HIT (86) web server and clustered at 97% sequence similarity to estimate the number of sequences.

The above-described method of direct plating resulted in a large amount of diversity from the *Populus* microbiome; however, this method likely favors fast-growing and abundant strains. To cultivate slow-growing or rare members, a cell-sorting approach was used. Three strains in the collection, Terriglobus albidus strain ORNL, *Roseimicrobium* sp. strain ORNL1, and *Starkeya* sp. strain ORNL1, were isolated through cell-sorting methods. Surface roots (approximately 5 inches deep) and associated soil (20 g total) were collected from a mature, wild *Populus deltoides* on the ORNL reservation, and 50 ml liquid R2A medium was added and the slurry was rocked at room temperature for 1 h. The suspension was filtered through sterile miracloth, and the liquid was centrifuged at 500 × *g* for 5 min to remove large particles and then passed through a 70-μm sieve. The filtrate was then centrifuged at 10,000 × *g*, 18°C, for 10 min to pellet the microbial cells. The pellet was gently resuspended in 4 ml R2A medium, overlaid on top of a Histodenz (Sigma-Aldrich, St. Louis, MO) solution (4 g Histodenz dissolved in 5 ml R2A) in 10-ml ultraclear centrifuge tubes (Beckman, Palo Alto, CA), and centrifuged at 10,000 × *g* for 40 min at 18°C (Optima LE-80K; Beckman Coulter, Brea, CA), as we previously described (74). The microbial white fraction that formed at the two-phase interface was recovered with a pipette and diluted in R2A medium for flow cytometric cell separation. The microbial sample was stained with 5 μM Syto 9 nucleic acid stain (Life Technologies, Grand Island, NY). The stained samples were sorted on a Cytopeia Influx cell sorter (BD, Franklin Lakes, NJ), with sorting gates selected based on relative particle size (forward scatter) and fluorescence intensity. Single particles were arrayed on petri plates containing R2A agar or DSMZ medium 1426 at a density of 100 particles per plate. Following incubation at 28°C for 5 to 10 days, colonies were screened by amplification of small subunit rRNA genes with bacterial universal primers (27F-1492R), followed by Sanger sequencing. A colony of Terriglobus albidus was identified on R2A agar with cells sorted from a low-fluorescence, small-particle-size gate (P1; Fig. S6). A colony of *Roseimicrobium* strain ORNL1 was identified on DSMZ medium 1426 plates sorted with large, high-fluorescence cells (gate P12; Fig. S6). A more detailed description of the microbial cultivation results using cell sorting will be published elsewhere. The genomes of Terriglobus albidus strain ORNL, *Roseimicrobium* strain ORNL1, and *Starkeya* sp. strain ORNL1 have been completed and published (41, 42, 53).

10.1128/mSystems.01306-20.6 FIG S6 Flow cytometry plot (forward scatter, FSC, versus green fluorescence, in relative units) of purified rhizosphere bacteria stained with SYTO9 dye. The gate areas (P1 and P2) were selected for single-cell sorting on agar medium plates. Download FIG S6, TIF file, 0.7 MB. Copyright © 2021 Carper et al. 2021 Carper et al. https://creativecommons.org/licenses/by/4.0/ This content is distributed under the terms of the Creative Commons Attribution 4.0 International license.

A 16S rRNA alignment was created from all the isolates using SINA (87) with the SILVA 132 alignment (88) as a reference. A cyanobacteria (Prochlorococcus marinus NR_125480.1) was used as the outgroup, since it resides outside all the isolates phylogenetically (89). Maximum likelihood phylogenetic inference was performed using IQ-TREE multicore (v1.6.8) (90). The best-fit model (TIMe+R16) for phylogenetic inference was selected based on Bayesian information criterion using ModelFinder (91) packaged with IQ-TREE. Alignment sites containing only gaps and/or Ns were removed before model selection and phylogenetic inference using FAST (92). The final tree was imported into ggtree (93) to add taxonomic ranks and metadata.

### Culture collection representativeness.

To ascertain how much of the natural poplar microbiome has been successfully cultivated, we obtained 16S rRNA sequencing data from two previous poplar studies, referred to as the common garden (NCBI SRA BioProject no. PRJNA666202) and Atlas (15). The Atlas study was conducted at a site in Blount County, Tennessee, with transitions from horizon silt loams to horizon silt clay loams. Five clonal individuals of *Populus deltoides* and *P. trichocarpa* x *deltoides* hybrid were sampled in August 2014. Further site and sampling description are detailed in reference 15. The common garden was set up as follows. Bulk soil, rhizosphere, and endosphere samples were collected from a diverse set of 32 genotypes of *P. trichocarpa* from two common gardens (Clatskanie, OR, and Corvalis, OR). These genotypes were selected to represent a wide range of phenotypic diversity (e.g., lignin%, S:G ratio, growth rate, lectin content, etc.). Sample preparation and DNA extraction were processed as described previously (18). DNA extracts were sent to JGI for sequencing. We trimmed the raw sequences to remove primers using cutadapt (v.1.18) (94). The sequences were imported into QIIME2 (v. 2019.1) (95) for further processing. Sequence variants were assigned using DADA2 (96) implemented in the QIIME2 plugin. Since multiple sequencing runs were performed, sequence variants were determined for each sequencing run and then combined per the DADA2 software recommendations. Taxonomy was assigned to the sequence variants using a naive Bayesian classifier trained on the SILVA 132 database (88) via the q2-feature-classifier (97). Sequence variants identified as chloroplast, mitochondria, or unassigned were removed before further analysis. The remaining sequence variants were reassigned a consensus taxonomy using the vsearch (98) plugin in QIIME2 with the culture collection 16S rRNA as the reference database. To determine if the top sequence variants were represented in the culture collection, we graphed the top 100 sequence variants using their relative abundance and relative frequency. Relative frequency was defined as the number of plant samples the sequence variant was present in divided by the total number of plant samples. All figures were created using ggplot2 (v 3.0.0) (99) in R (100).

The 16S rRNA gene sequences from Acinetobacter species were downloaded from NCBI, and Psychrobacter arcticus (NR_042907.1) was used as an outgroup. The Acinetobacter 16 rRNA sequences, including the top sequence variant from the common garden and the isolate strain, were aligned using SINA (87) with the SILVA 132 alignment (88) as a reference.

Maximum likelihood phylogenetic inference was performed using IQ-TREE multicore (v1.6.8) (90). The best-fit model (TIMe+R16) for phylogenetic inference was selected based on Bayesian information criterion using ModelFinder (91) packaged with IQ-TREE. Alignment sites containing only gaps and/or Ns were removed before model selection and phylogenetic inference using FAST (92). The final tree was imported into ggtree (93).

### Constructed communities.

One 10-strain bacterial community was created from the culture collection from *Populus deltoides*. The 10-member community was made at the phylum taxonomic rank to resemble the distribution seen in the natural community of wild poplar. The 10 members included Streptomyces mirabilis YR139 (*Actinobacteria*), *Bacilius* sp. strain BC15 (*Firmicutes*), *Sphingobium* sp. strain AP49 (*Alphaproteobacteria*), *Caulobacter* sp. strain AP07 (*Alphaproteobacteria*), *Rhizobium* sp. strain CF142 (*Alphaproteobacteria*), *Paraburkholderia* sp. strain BT03 (*Betaproteobacteria*), *Variovorax* sp. strain CF313 (*Betaproteobacteria*), *Duganella* sp. strain CF402 (*Betaproteobacteria*), Pseudomonas sp. strain GM17 (*Gammaproteobacteria*), and *Pantoea* sp. strain YR343 (*Gammaproteobacteria*). Genome annotations using KEGG orthology were used to compare to *P. deltoides* microbiome metagenome (IMG accession number 3300006177) (45). Specifically, KO terms represented in the 10-member community were compared directly to the list of KO terms identified in the representative metagenome for presence/absence. The 10-member community from *Populus deltoides* will furthermore be referenced as PD10. Three experiments were performed using the PD10 community. For all experiments on *Populus*, the initial inoculum was prepared as described below. To prepare the community for inoculation, strains were grown in 5 ml of R2A medium overnight at 25°C and 200-rpm shaking. Bacterial suspensions were washed with 10 mM MgSO_4_ and then diluted to an optical density at 600 nm (OD_600_) of 0.01. We combined 1 ml of each strain, and 10 ml total of inoculum was added to the plant soil immediately prior to planting. Plant culturing and inoculation were previously described (37).

The first experiment examined the PD10 community assemblage over time in *P. trichocarpa* root tissue. The PD10 community was grown as described above and inoculated into the soil before planting of *P. trichocarpa* axenic plants. Ten plants were used for each time point, 5 uninoculated controls and 5 inoculated plants. Root tissue was harvested at 1, 7, 14, and 21 days postinoculation. DNA was extracted as described above. The 16S rRNA gene was amplified for both the cultures and root tissues and sequenced using the Illumina protocol described in reference 15. Raw amplicon reads were processed as described above through QIIME 2. Taxonomy was also assigned using the consensus vsearch (98) option in QIIME2 against a database of 16S sequences from the PD10 community members. The resulting sequence variant table, mapping file, and taxonomy file were imported into Phyloseq (version 1.22.3) (101) in R (version 3.4.4) (100) for visualization. We corrected for 16S rRNA copy number using a custom R script (available at https://github.com/dlcarper/CopyNumberCorrection) and the number of copies obtained from the isolate genomes. Raw sequences were deposited in the NCBI SRA database under BioProject number PRJNA659670.

The second experiment was single inoculations of each of the PD10 members onto axenic *Populus* roots, and colonization of roots was assessed by counting the number of CFU for each strain individually. Plants were removed from the microcosm. Roots were submerged in sterile distilled water to remove the clay from the root system. The wet weight of plant root tissue was recorded, and root tissue was macerated with 10 mM MgSO_4_ at 1 ml per g of root tissue. Macerated sample was serially diluted (10-fold) with 10 mM MgSO_4_. Each sample was plated onto R2A medium plates and allowed to grow for 48 h at 25°C, after which the colonies were counted.

Finally, in the third experiment, the PD10 community was inoculated onto axenic *Populus delotides* and *Populus trichocarpa* roots, and the final community was evaluated using qPCR. Two plant species were used for inoculation, *P. deltoides* (genotype WV94) and *P. trichocarpa* (genotype BESC-819) to test for differences in host selectivity. Five plants from *P. deltoides* and *P. trichocarpa* were inoculated with PD10 communities. Plants were grown for 21 days in growth chambers under 16 h light, 8 h dark per day with ∼50% humidity. The plants were harvested by removing the entire plant from the soil and rinsing the roots with sterile water to remove loose soil. Root material was flash frozen in liquid nitrogen and stored at −80°C. Tissue lysis was obtained using three rounds of alternating bead beating (1 min) and liquid nitrogen freezing, followed by extraction using the PowerPlant kit according to the manufacturer’s instructions. DNA was used for microbial quantification by qPCR. qPCR for bacterial quantification was performed using the iTAQ kit (Bio-Rad, Hercules, CA, USA) on a CFX96 system according to manufacturer’s instructions using bacterial genomic DNA as a standard for quantification, as described previously (37).

### Data availability.

Raw sequences for the common garden data set were deposited in the NCBI SRA database under BioProject number PRJNA666202. Raw sequences for the constructed communities were deposited in the NCBI SRA database under BioProject number PRJNA659670 for poplar data.
