# Supplementary figures and images for: Cultivating the Bacterial Microbiota of Populus Roots
Source: mSystems. 2021 Jun 22;6(3):e01306-20. doi: 10.1128/mSystems.01306-20 (PMC8269261; doi:10.1128/mSystems.01306-20)

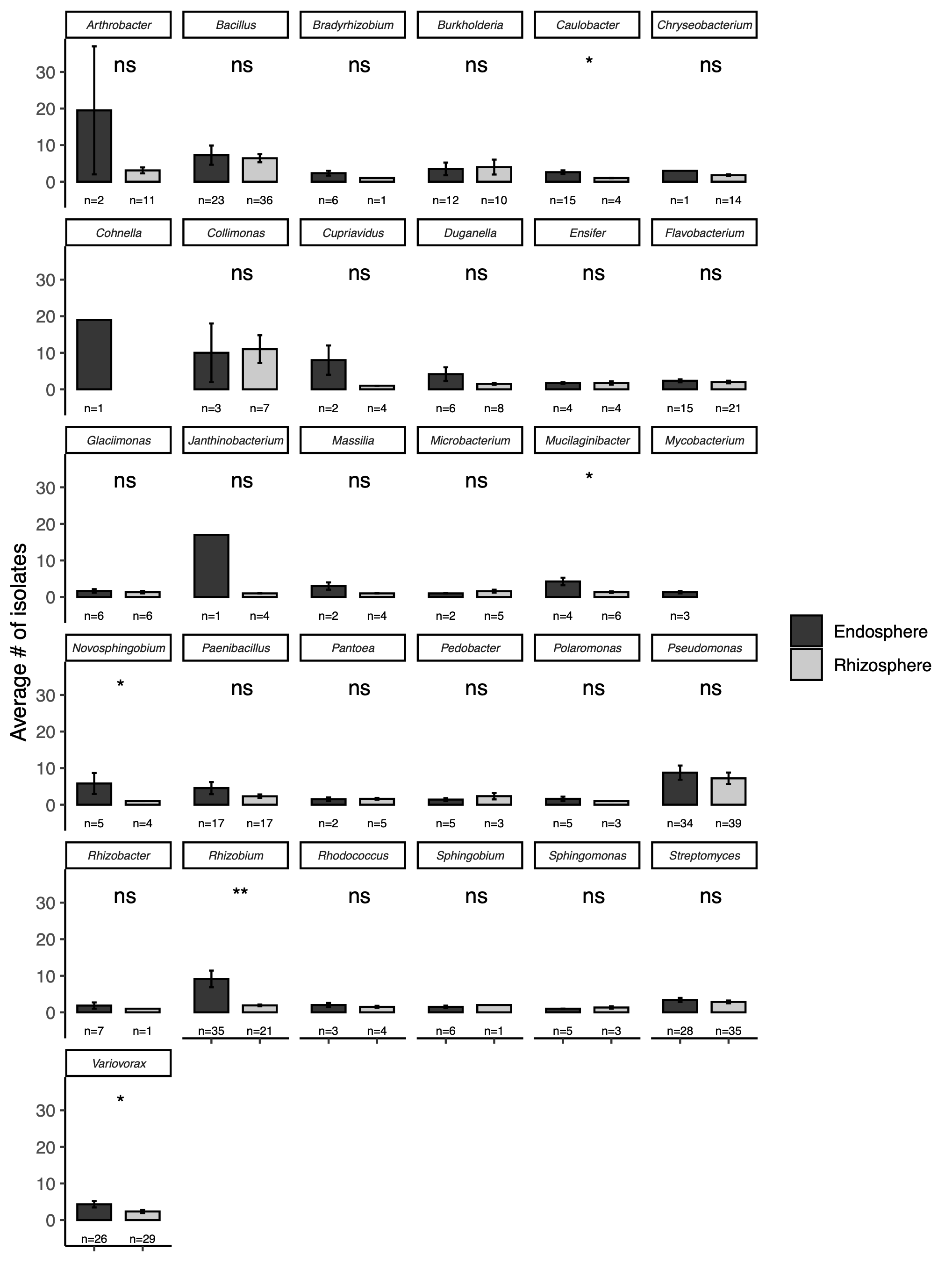

Supplement: FIG S1 [file msystems.01306-20-sf001.tif]

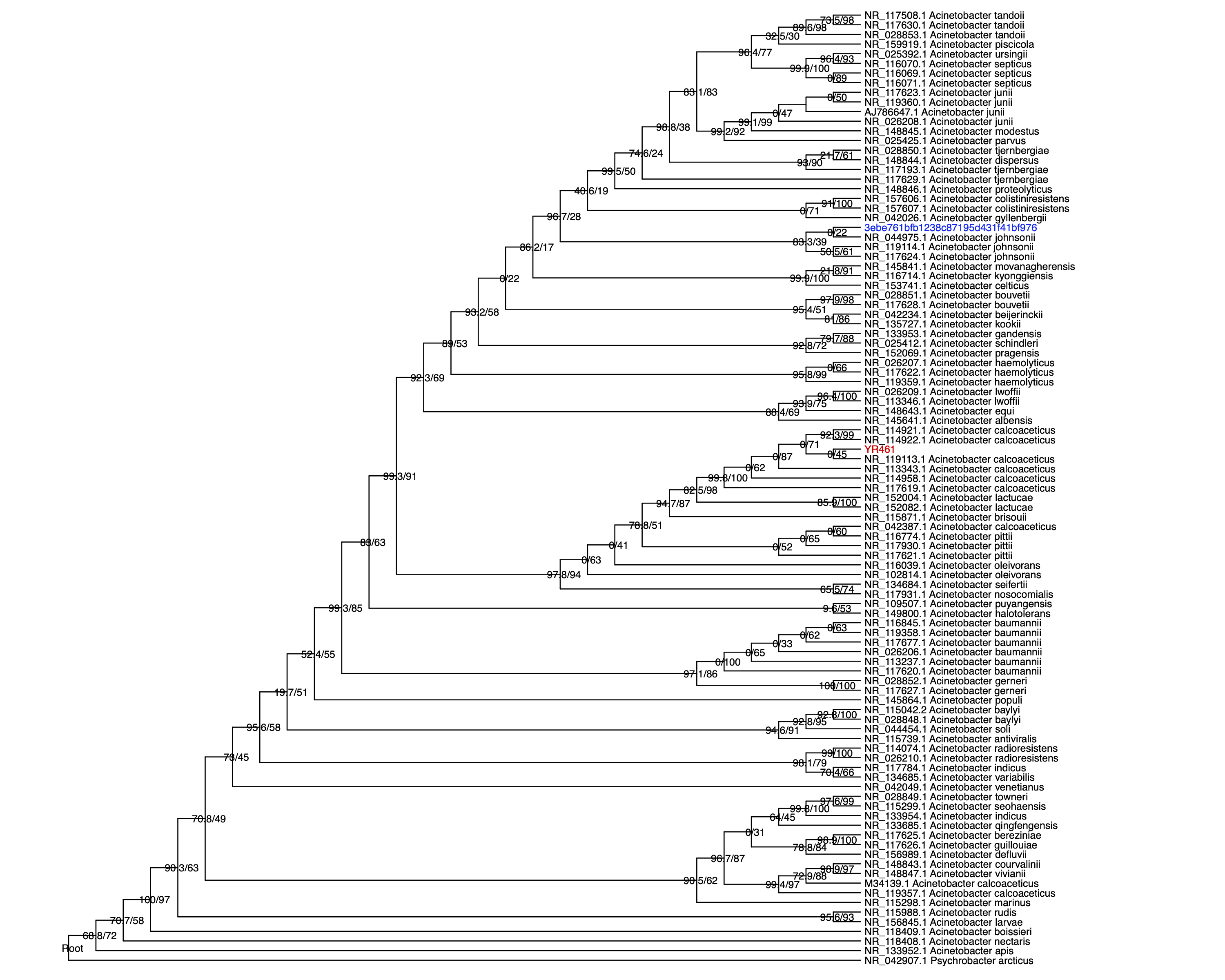

Supplement: FIG S2 [file msystems.01306-20-sf002.tif]

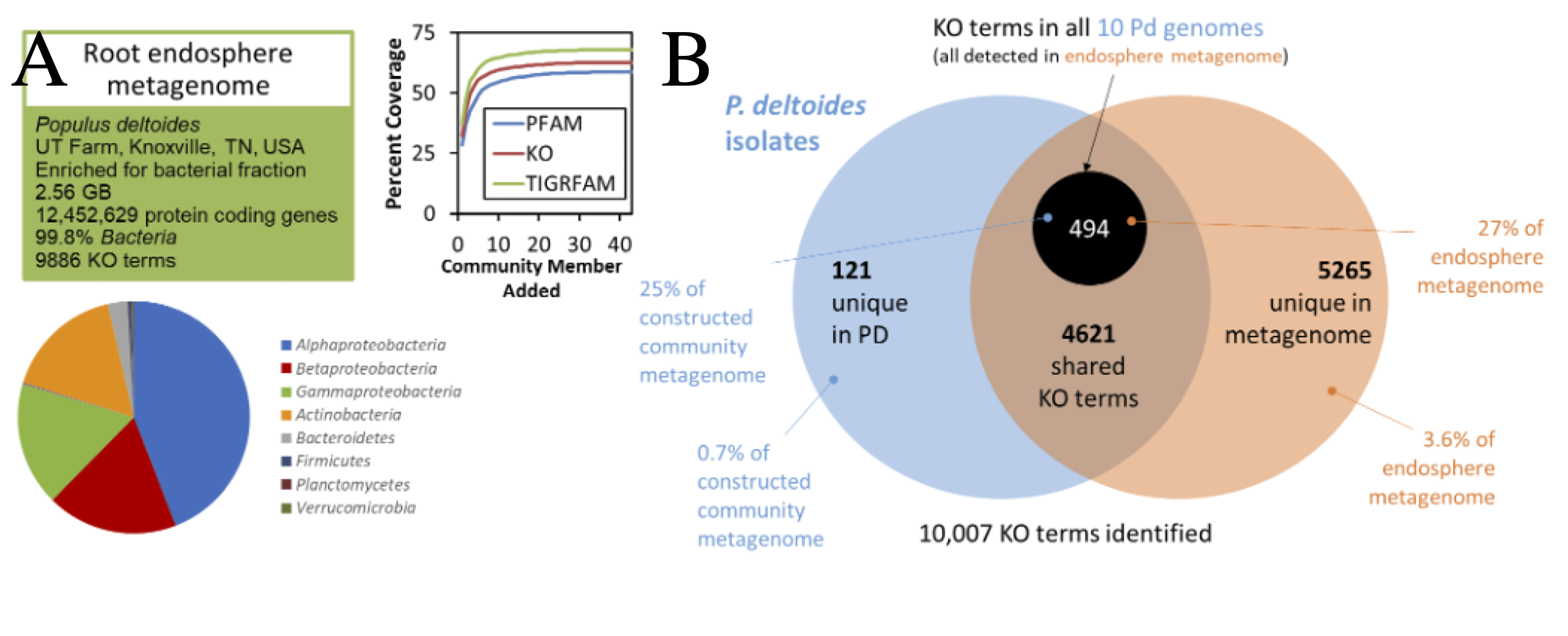

Supplement: FIG S3 [file msystems.01306-20-sf003.tif]

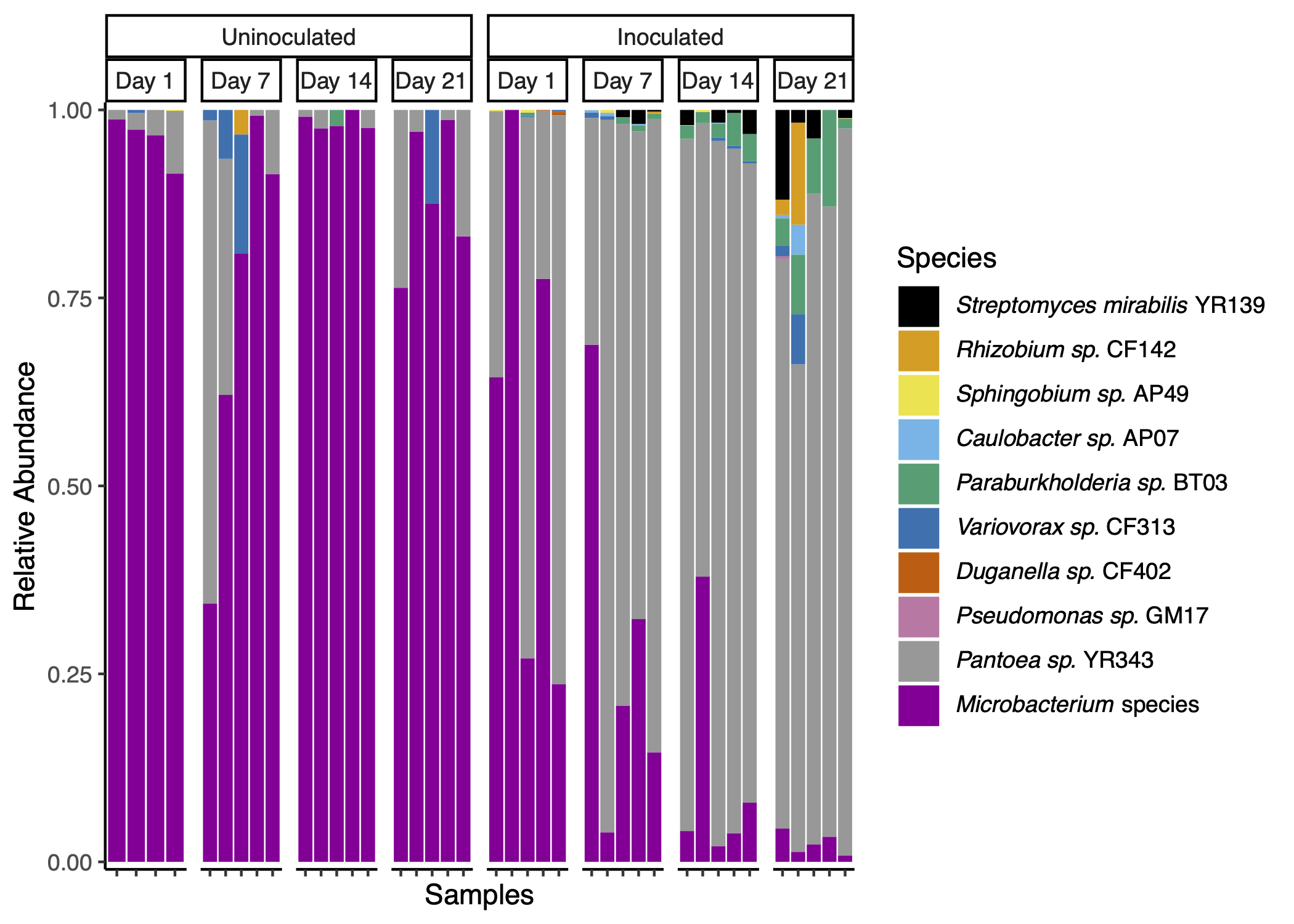

Supplement: FIG S4 [file msystems.01306-20-sf004.tif]

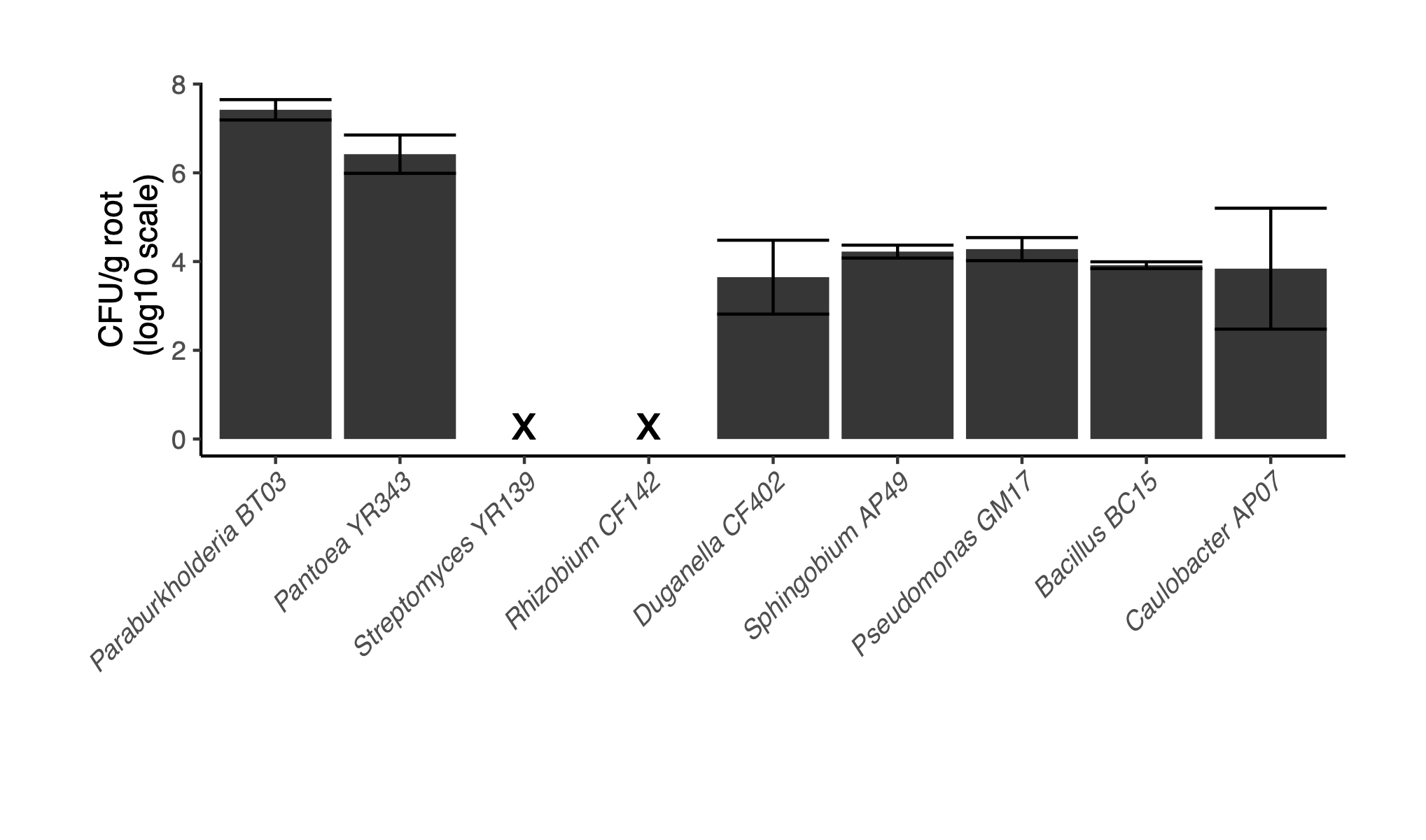

Supplement: FIG S5 [file msystems.01306-20-sf005.tif]

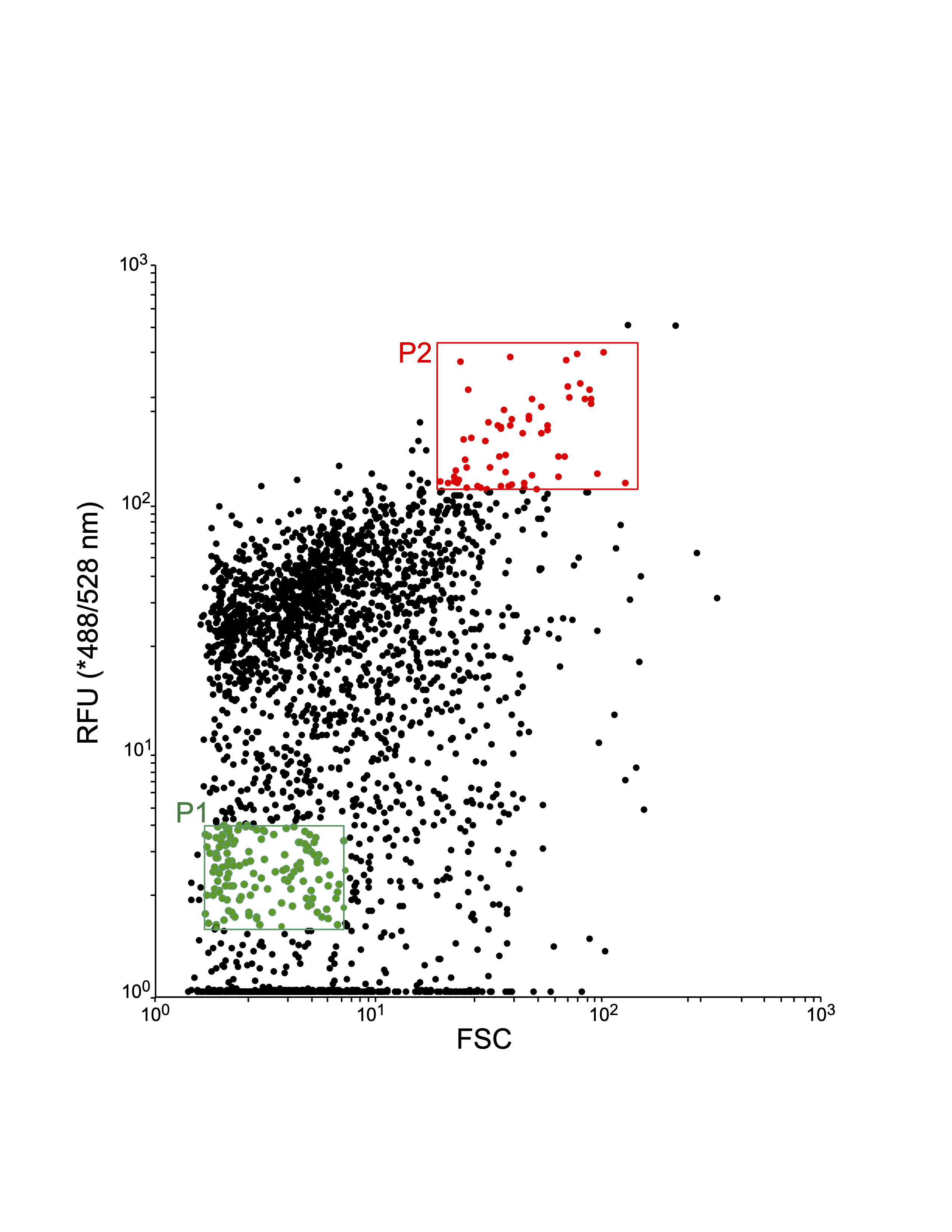

Supplement: FIG S6 [file msystems.01306-20-sf006.tif]
